# Hemodynamic changes and their relationship with white matter hyperintensities in CSVD patients with cognitive impairment: a 4D flow study

**DOI:** 10.3389/fnagi.2025.1578288

**Published:** 2025-06-18

**Authors:** Jiajun Cao, Chang Yuan, Zhenpeng Zhang, Xiaoying Zhao, Yukun Zhang, Peipei Chang, Qingwei Song, Bingwei Zhang, Rui Hu, Yanwei Miao

**Affiliations:** ^1^Department of Radiology, The First Affiliated Hospital of Dalian Medical University, Dalian, China; ^2^Shanghai Medical College, Fudan University, Shanghai, China; ^3^Department of Neurology, The First Affiliated Hospital of Dalian Medical University, Dalian, China; ^4^Department of Radiology, Taihe Hospital, Hubei University of Medicine, Hubei, China

**Keywords:** 4D flow MRI, cerebral small vessel disease, cognitive impairment, white matter hyperintensities, hemodynamics

## Abstract

**Objective:**

To observe the hemodynamics of intracranial arteries and veins in patients with cerebral small vessel disease (CSVD) with cognitive impairment (CI), and to explore the association between these flow features and white matter hyperintensities (WMH).

**Materials and methods:**

A total of 53 patients with CSVD were included in the study, comprising 30 patients with CI (CI group) and 23 patients with non-CI (NCI group); Meanwhile, 25 age-matched cognitively healthy volunteers were recruited. WMH burden was evaluated using a 2D axial T2-FLAIR sequence. A 4D flow MRI was employed to measure intracranial hemodynamic features, including cross-sectional area, flow rate, blood flow velocity, wall shear stress (WSS), pulsatility index, and resistive index in the internal carotid artery (ICA), middle cerebral artery, basilar artery (BA), transverse sinus (TS), straight sinus (SS), and superior sagittal sinus (SSS). CSF-Q flow, a 2D PC MRI sequence, was performed to calculate the CSF fluid dynamics in the midbrain aqueduct.

**Results:**

The CSVD with CI population reported a statistically significant decrease in flow rate, blood flow velocity, and WSS, as well as an increase in PI, RI, CSF flow quantity, and velocity compared to age-matched cognitively healthy control participants. There was a moderately positive correlation between MMSE, MoCA score and flow rate, flow velocity, and WSS (*r* = 0.226–0.544, all *P* < 0.05), and a moderately negative correlation between MMSE, MoCA score and PI, RI (*r* = −0.230 to −0.406, all *P* < 0.05). Multiple linear regression indicated that, the flow rate and mean velocity in venous sinuses (β = −0.472 to −0.381, all *P* < 0.05) and the WSS in arterial segments (β = −0.771 to −0.441, all *P* < 0.05) had independently negative association with WMH burden; Meanwhile, a significant positive relationship was found between PI in arterial segments and specific-distributed WMH (PVWMH and S-CC WMH) (β = 0.239 to 0.356, all *P* < 0.05).

**Conclusion:**

The intracranial hemodynamics were associated with CI and WMH in patients with CSVD. 4D flow MRI can be used as a non-invasive method to assess cerebrovascular hemodynamics and helps to identify patients who may benefit from interventions to improve the functions of the cerebral circulatory system and provides a potential new path for clinical treatment.

## 1 Introduction

Cerebral small vessel disease (CSVD) associated with age and vascular risk factors has been the main cause of vascular cognitive impairment (VCI) (Yang et al., 2022; Smith and Beaudin, 2018). Based on the neuropathological findings, the accompanying brain vascular and degenerative damage increases the risk of dementia by more than twofold, thus representing the dominant cause of CI in the elderly (Dobrynina et al., 2020; Azarpazhooh et al., 2017). VCI-related tissue damage is more common in white matter, where the histopathology of injury includes both focal and diffuse lesions, often presenting in various combinations and proportions. Focal lesions are regarded as the result of an acute response to local ischemia, while diffuse white matter lesions are considered adaptations to abnormal perfusion and physiological conditions in the tissue. Therefore, the changes in white matter may partly reflect the progressive pathological response of normal white matter to hemodynamic changes, and white matter hyperintensities (WMH) may be a late result of this white matter damage (Englund, 2002; Chen et al., 2021; de Groot et al., 2013). As an easily observable imaging manifestation, WMH is currently considered to be one of the strongest predictors of cognitive performance and has a stronger correlation with specific cognitive domains, such as attention, executive function and information processing speed (Alber et al., 2019; Chen et al., 2018). Studies have shown that a larger WMH volume is associated with slower cerebral local network transfer efficiency and information processing speed (Hilal et al., 2021). Moreover, in the white matter tracts that are important for information processing, a higher WMH load is associated with slower cognitive speed (Vergoossen et al., 2020). According to their location, WMH can usually be divided into periventricular white matter hyperintensities (PVWMH) and deep white matter hyperintensities (DWMH). DWMH is correlated with attention, executive function, and visuospatial function, while PWMH is related to all cognitive functional domains (Sun et al., 2022; Su et al., 2022; Li et al., 2021). Besides the morphological changes observed by MRI, evidence suggested that the pathological changes of the vascular system may play a role in cognitive impairment associated with cerebral small vessel disease, lower cerebral blood flow predicted cognitive decline in patients with VCI (van Dinther et al., 2023). The changes of cerebrospinal fluid (CSF) dynamics in midbrain aqueduct were possibly associated with greater CSVD burden, and the abnormality of intracranial hemodynamics, such as greater pulsatility index in arteries and venous sinuses may contribute to the CSVD (Shi et al., 2018a; Vikner et al., 2022; van Tuijl et al., 2022). Hemodynamics is closely related to the CSF flow, however, no study has been conducted to comprehensively investigate the cerebral hemodynamics (both in arteries and venous) and CSF dynamics in CSVD patients with CI. Consequently, there is interest in non-invasive methods to assess global cerebrovascular hemodynamics as potential systemic pathogenic or exacerbating factors in CSVD and potential avenues for treatment.

Transcranial Doppler (TCD) ultrasound and 2D phase contrast (PC) MRI are two different commonly used methods for probing intracranial hemodynamic parameters. However, TCD has limited ability to detect the distal branches of intracranial blood vessels. Due to the flat structure of midbrain aqueduct on the MRI images, it is easy to locate by 2D PC MRI sequence, and the short acquisition time and relatively simple post-processing lead the CSF-Q flow sequence become a potential high-throughput method to evaluate the CSF dynamics at the midbrain aqueduct in clinical practice (Battal et al., 2011; Bessen et al., 2023). Because of its sufficient spatial and temporal resolution, 4D flow MRI is very suitable for comprehensive cerebral hemodynamic evaluation. By encoding velocity in three directions, 4D flow MRI can retrospectively quantify blood flow at any position within the scanned volume. In our previous study, we used compressed sensing technology (CS) to accelerate the acquisition of 4D flow MRI, which shortened the scanning time and improved its applicability in clinical situations while ensuring quantitative accuracy (Cao et al., 2024).

Therefore, the purpose of the present study was to conduct a comprehensive assessment of the cerebral vasculature of CSVD patients with CI to explore the association between these flow indicators and the distribution and intensity of WMH. We hypothesized that CSVD patients with CI exhibit distinct hemodynamic patterns compared to controls, and these abnormalities correlated with cognitive function, WMH distribution and severity. In the study, we used CS-accelerated 4D flow MRI to quantify the hemodynamics of internal carotid artery (ICA) (C2, C4, C7 segments), middle cerebral artery (M1 segment), basilar artery (BA), transverse sinus (TS), straight sinus (SS), and superior sagittal sinus (SSS). Meanwhile, 2D PC MRI was used to evaluate the fluid dynamics of CSF at the midbrain aqueduct.

## 2 Materials and methods

### 2.1 Participants

CSVD patients (aged from 50 to 80 years) were recruited from The First Affiliated Hospital of Dalian Medical University based on the following inclusion criteria (Wardlaw et al., 2013b): (1) the clinical manifestations of CSVD, including lacunar stroke syndrome with symptoms lasting more than 24 h that occurred more than 6 months prior to the visit; or TIA with a duration of < 24 h accompanied limb weakness, semi-sensory loss, or dysarthria occurring more than 6 months previously; (2) Conformity to the neuroimaging diagnostic criteria of CSVD, including the WMH, recent small subcortical infarcts, lacunes, perivascular spaces, microbleeds, and brain atrophy; (3) normal laboratory examinations, including blood biochemical indices and liver and kidney function tests; (4) Complete imaging data; (5) No treatment was performed before MRI scanning. Exclusion criteria included: (1) patients with vascular occlusion or severe stenosis that significantly affect hemodynamic analysis or cerebral vascular dysplasia; (2) severe dementia or cognitive dysfunction caused by other conditions, such as Alzheimer's disease; (3) patients with brain tumors or other systemic malignant tumors, a history of traumatic brain injury, acute massive cerebral infarction, or craniocerebral surgery; (4) liver, kidney, heart, lung, or other important organ dysfunction; (5) cerebral hemorrhage.

### 2.2 Cognitive assessment

A trained neurology physician performed a brief medical history (including a medication review) and physical examination of the patients. Within 1 week of MRI scan, two experienced neurology physicians evaluated the overall cognitive statuses of the patients by using mini mental state examination (MMSE) and the Montreal cognitive assessment (MoCA), the results were determined by the two physicians after consultation. To mitigate practice effects, MoCA and MMSE were administered 1 week apart. Both neurocognitive rating scales were administered in their Chinese versions to match the participants' native language. The results of the MMSE and MoCA were used to determine whether the participants had cognitive impairment (CI) (Wu et al., 2022; Nijsse et al., 2016). The specific standards were as follows: a MMSE score < 27 and/or the MoCA score < 26 (with years of education ≤ 12 years receiving an additional point) were considered to have CI. Based on the cognitive assessments, patients with dementia were excluded.

### 2.3 MRI protocol

All participants underwent MR examination using a 3.0 T MR scanner (Ingenia CX, Philips Healthcare, Best, Netherlands) equipped with a 32-channel head coil. The 4D flow sequence was acquired with CS acceleration to quantify the hemodynamics of intracranial arteries and venous sinuses. The imaging parameters were as follows: repetition time (TR)/echo time (TE) = 5.1/3.0 ms; flip angle = 8°; field of view (FOV) = 180 × 180 × 46 mm^3^; acquisition voxel size = 1.6 × 1.6 × 1.6 mm^3^; reconstruction voxel size = 0.8 × 0.8 × 0.8 mm^3^; acquisition matrix = 112 × 112; reconstruction matrix = 224 × 224; and velocity encoding (VENC) = 80/60 cm/s (cerebral artery/venous sinus); CS acceleration factor = 4; the scan time of a single 4D flow sequence = 5 min 59 s (taking heart rate of 60 beats per minute and the number of slices = 58 as an example). The CSF-Q flow sequence was used for analyzing CSF fluid dynamics. The slice perpendicular to the midbrain aqueduct between the superior and inferior colliculi of the midbrain was selected as its quantitative analysis plane, with the following imaging parameters: TR/TE = 12/7.4 ms; FOV = 150 × 150 × 4 mm^3^; voxel size = 0.59 × 0.84 × 4.00 mm^3^; flip angle = 15°; VENC=15 cm/s. The 4D flow MRI and CSF-Q flow scans were acquired with retrospective ECG-triggering (with 20 retrospectively reconstructed cardiac phases for 4D flow MRI, 16 for CSF- Q flow). 2D axial T2 fluid-attenuated inversion recovery sequence (T2-FLAIR) was used to observe and evaluate the WMH, with the following imaging parameters: TR/TE = 9,000/125 ms; inversion recovery time (TI) = 2,500 ms; FOV = 230 × 187 × 149 mm^3^; voxel size = 0.75 × 1.04 × 6.5 mm^3^.

### 2.4 Image processing and analysis

#### 2.4.1 Evaluation of WMH

**Figure 1:**
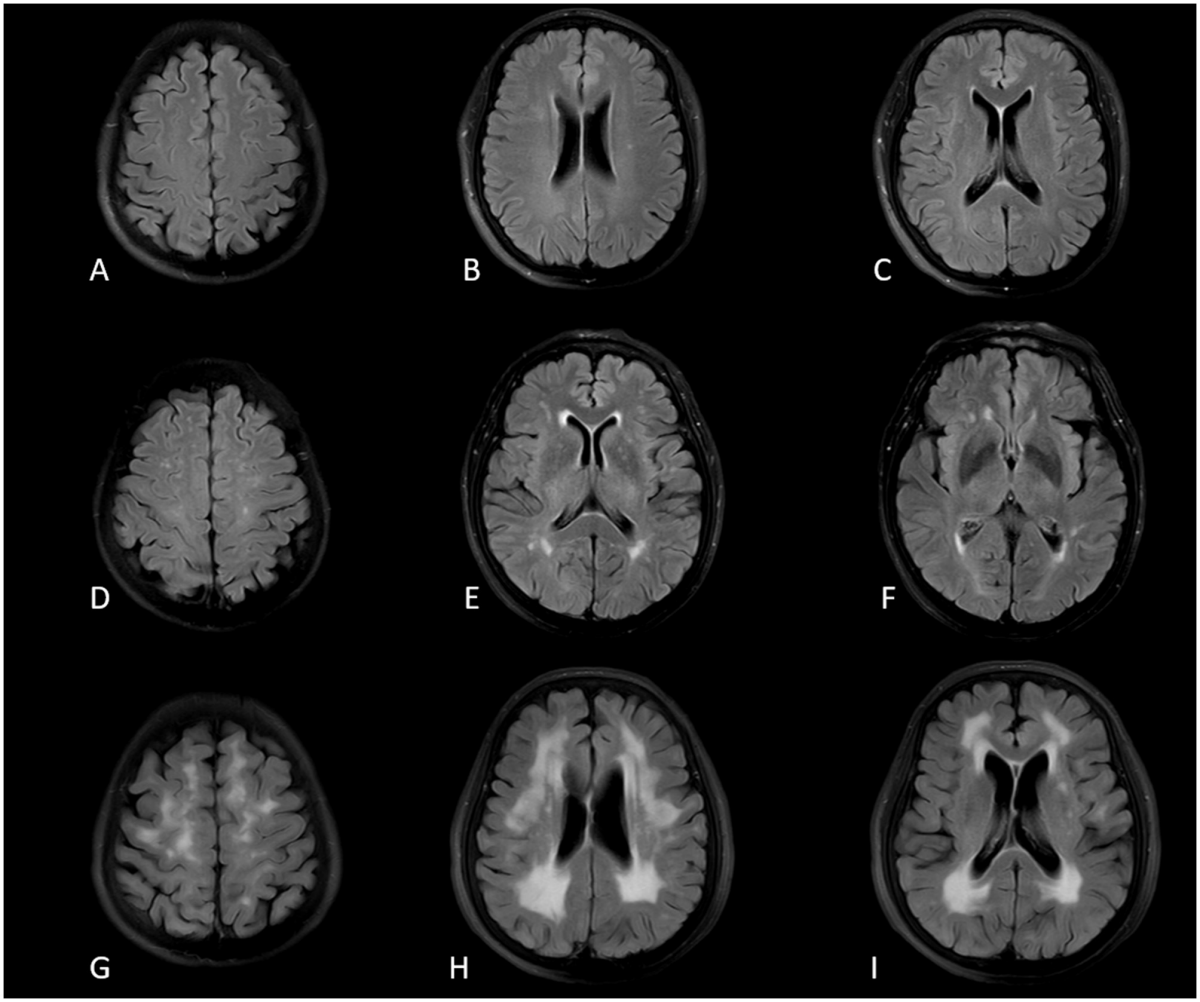
Fazekas classification schematics for PVWMH and DWMH on T2-FLAIR images. **(A–C)** Fazekas grade 1; **(D–F)** Fazekas grade 2; **(G–I)** Fazekas grade 3.

Two experienced neuroimaging physicians observed and evaluated the degree of WMH according to the Fazekas classification (Fazekas et al., 2002), The results were determined by the two observers after consultation: (1) periventricular white matter hyperintensities (PVWMH): 0 = no lesions, 1 = thin layer lesions, 2 = smooth halo-like lesions, 3 = irregular periventricular high signal extends to deep white matter; (2) deep white matter hyperintensities (DWMH): 0 = no lesions, 1 = punctuate lesions, 2 = the lesions began to fuse, scattered plaque-like, 3 = the lesions were large area fusion, patchy-like (Figure 1). The Fazekas grade was the sum of PVWMH and DWMH scores to describe the severity of white matter hyperintensity in the whole brain. In addition, the splenium of the corpus callosum WMH (S-CC WMH) as a special location, adjacent to the ventricles of the WMH type (Garnier-Crussard et al., 2021), its evaluation standard referred to the above PVWMH evaluation criteria.

#### 2.4.2 Hemodynamics analysis: arteries and venous sinuses

**Figure 2:**
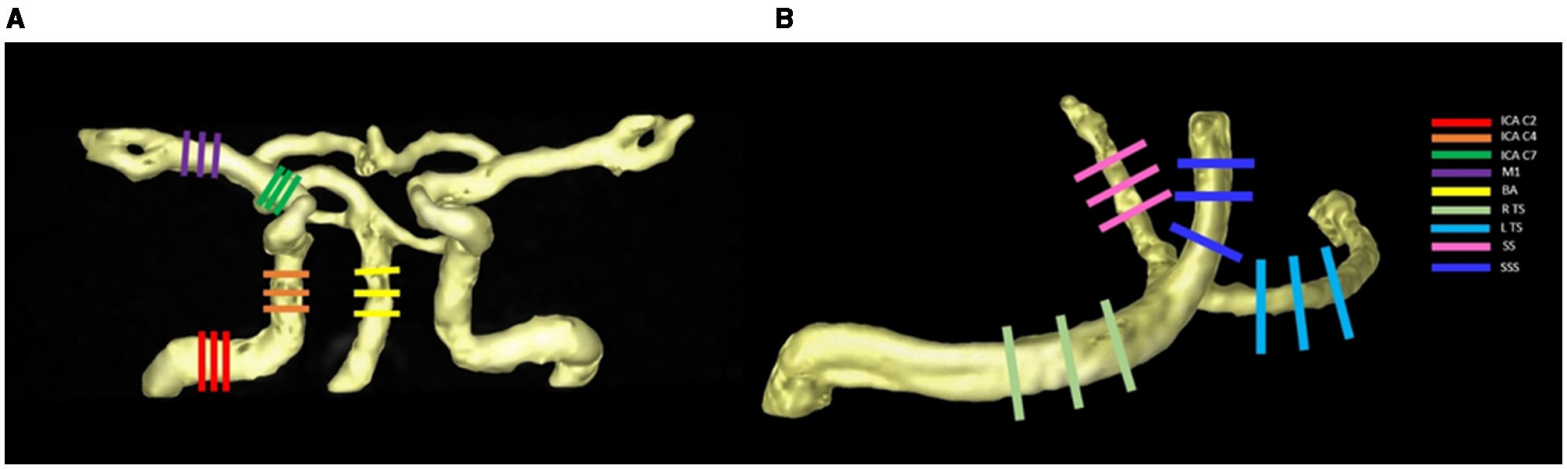
Selected arterial segments (using the right side of internal carotid artery and middle cerebral artery as an example) **(A)** and venous sinuses **(B)** with measurement planes placed perpendicular to the vessel orientations.

The hemodynamic analysis method is consistent with our previous study using the cvi42 software (version 5.14, Circle Cardiovascular Imaging, Canada) (Cao et al., 2024). The processing steps included data cropping, preprocessing, vessel segmentation, and hemodynamic measurement. The specific workflow was as follows: The 4D flow raw data, including magnitude image and phase image, were imported into the software. Firstly, we selected the region of interest that covers the target vessel to remove tissue outside the region of interest (e.g., air, static tissues). Secondly, the software automatically defined a static tissue mask, we could use the suggested mask for offset correction or adjust it and make sure that there were no unwanted regions included in the mask. Having flow or noise included in the static tissue could have a severe negative influence on the result. Additionally, we also performed Phase-Anti-Aliasing on those images with aliasing, which is also done automatically by the software. Thirdly, the PC MR angiography would be calculated by the software with a 3D volume rendering, and we used 3D cut and crop to remove excess noise generated by the static tissues and air probably to refine the vessel segmentation in the PC MR angiography. Finally, the hemodynamic parameters were obtained by placing the measurement plane orthogonal to the midline of the selected lumens. To account for the hemodynamic effect of physiological vascular tortuosity (Xie et al., 2024), nine arterial segments were selected: the bilateral internal carotid artery (ICA) C2, C4, C7 segments and middle cerebral artery (MCA) M1 segments, basilar artery (BA), and four venous sinuses were selected: the bilateral transverse sinus (TS), straight sinus (SS), and superior sagittal sinus (SSS) (Figure 2). The hemodynamics of each vessel were described by the mean of the three planes, including cross-sectional area, flow rate (The volume of blood flow through the measurement plane per unit time), mean/peak velocity (The mean/peak velocity of blood flow through the measurement plane per unit time), wall shear stress (WSS), pulsatility index [PI = (*Q*_max_ – *Q*_min_)/*Q*_mean_; *Q* = flow rate], and resistive index [RI = (*Q*_max_ – *Q*_min_)/*Q*_max_].

#### 2.4.3 CSF fluid dynamics analysis

**Figure 3:**
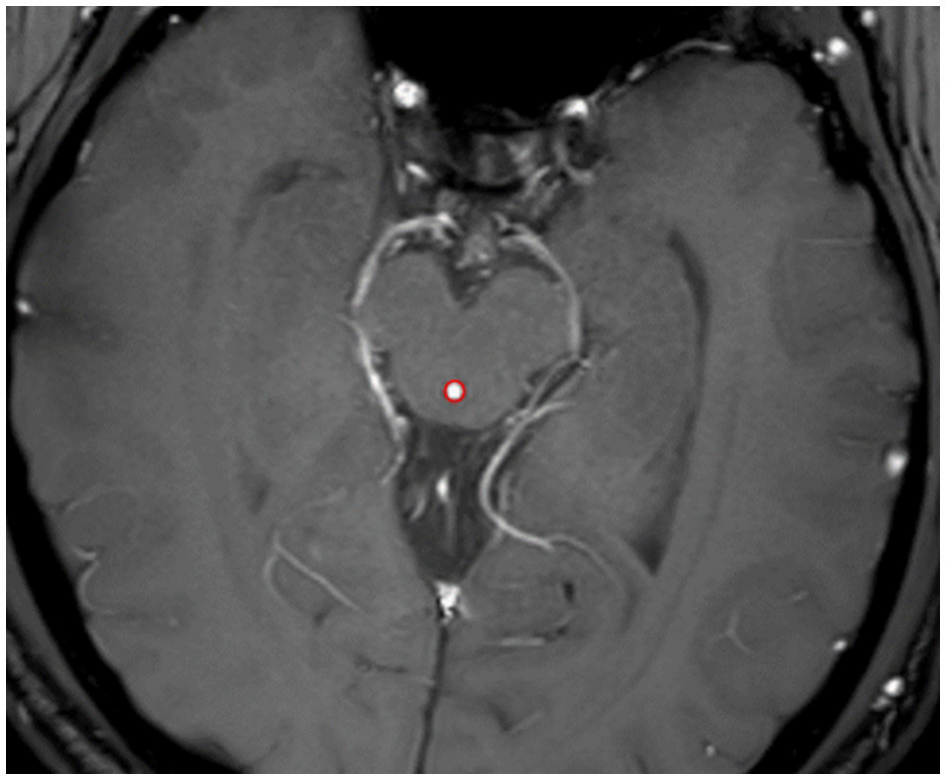
ROI placement diagram at midbrain aqueduct (the red circle).

Quantitative analysis of CSF-Q Flow images was performed using Q Flow analysis module of the Philips IntelliSpace Portal (ISP). The window width and window level were adjusted to clearly display the midbrain aqueduct and to place a suitable circular region of interest (ROI) with an average size of 2–4 mm^2^ (Figure 3). The software automatically analyzed the flow data in the ROI of each frame image and obtained the area, flux (The volume of CSF flow through the ROI section per unit time), and mean/peak velocity (The mean/peak velocity of CSF flow through the ROI section per unit time) of the head-to-foot side and the foot-to-head sides of CSF during a cardiac cycle for each frame.

### 2.5 Statistical analysis

Statistical analyses were conducted using SPSS version 26.0. A Shapiro–Wilk test was used to evaluate normality. The results are expressed as mean ± SD if the parameters were normally distributed and as median (interquartile range) if the parameters had a skewed distribution. An ANOVA test was used for normally distributed measurement data and the pairwise differences between groups were assessed using LSD-t tests. Non-normally distributed continuous variables were analyzed using Kruskal–Wallis H-tests, with a Dunn-Bonferroni test for *post hoc* comparisons. Chi square test was used to access differences in sex, prevalence of vascular risk factors (hypertension, hyperlipidemia, diabetes), unhealthy habits (ever smoking history and ever drinking history) among different groups. Spearman correlation analysis was performed between hemodynamics parameters and the results of cognitive assessment (MMSE and MoCA). Multiple linear regression (controlled for the age, sex, BMI, education years, vascular risk factors, and unhealthy habits), with flow rate, mean velocity, WSS, and PI as the predictor variables and the severity of WMH as the dependent variables, was performed in the full cohort (*N* = 78). The statistical significance level was set at *P* < 0.05, two-tailed.

## 3 Results

### 3.1 Participant characteristics

A total of 78 participants were included, comprising 25 cases of healthy controls (HC group), 23 cases of CSVD with non-CI (NCI group), and 30 cases of CSVD with CI (CI group). There were no significant difference in sex (χ^2^ = 0.54, *P* = 0.765), age (*F* = 2.14, *P* = 0.125), years of education (*H* = 5.44, *P* = 0.066) and BMI (*F* = 0.19, *P* = 0.826) among the three groups. There was a significant difference in prevalence of hypertension (χ^2^ = 18.07, *P* < 0.001) among the groups. There were no significant difference in the prevalence of diabetes (χ^2^ = 1.63, *P* = 0.453), hyperlipidemia (χ^2^ = 4.58, *P* = 0.101) and smoking (χ^2^ = 0.53, *P* = 0.766), drinking (χ^2^ = 2.29, *P* = 0.319) among the groups. There was a significant difference in MMSE score (*H* = 33.55, *P* < 0.001), MoCA score (*H* = 56.09, *P* < 0.001), Fazekas grade (*H* = 59.05, *P* < 0.001), PVWMH (*H* = 59.39, *P* < 0.001), DWMH (*H* = 52.62, *P* < 0.001), and S-CC WMH grade (*H* = 32.96, *P* < 0.001) among the groups (Table 1).

**Table 1:** Characteristics of CSVD patients and the HCs.

**Parameters**	**HC (*N =* 25)**	**NCI (*N =* 23)**	**CI (*N =* 30)**	**Effect size (ES)**	***P***
Sex (male, female)	11,14	12,11	16,14	0.061	0.765
Age (years)	63.24 ± 5.97	65.04 ± 6.93	66.70 ± 5.74	0.054	0.125
Education (years)	12.00 (9.00,15.00)	12.00 (9.00,15.50)	9.00 (6.75,12.00)	0.045	0.066
BMI	24.28 ± 3.32	23.90 ± 2.92	24.36 ± 2.28	0.005	0.826
Hypertension (*n*, %)	5 (20%)	7 (30.4%)	22 (73.3%)^*^,Δ	2.046	< 0.001
Diabetes (*n*, %)	2 (8%)	4 (17.4%)	6 (20%)	0.182	0.453
Hyperlipidemia (*n*, %)	11 (44%)	8 (34.8%)	19 (63.3%)	0.518	0.101
Smoking (*n*, %)	8 (32%)	6 (26.1%)	7 (23.3%)	0.060	0.766
Drinking (*n*, %)	4 (16%)	7 (30.4%)	10 (33.3%)	0.259	0.319
MMSE score	28 (28.00,29.00)	28.00 (28.00,29.00)	25.00 (22.00,27.00)^*^,Δ	0.410	< 0.001
MoCA score	27.00 (26.00,28.00)	27.00 (26.00,28.00)	19.00 (14.50,22.25) ^*^,Δ	0.702	< 0.001
Fazekas grade	2 (1,2)	4 (4,6) ^*^	6 (5,6) ^*^,Δ	0.741	< 0.001
PVWMH	1 (1,1)	2 (2,3) ^*^	3 (3,3) ^*^,Δ	0.745	< 0.001
DWMH	1 (1,1)	2 (2,3) ^*^	3 (3,3) ^*^	0.658	< 0.001
S-CC WMH	0 (0,0)	1 (0,2) ^*^	2 (1,3) ^*^,Δ	0.402	< 0.001

^*^There was a significant statistical difference, compared to the HC group, P < 0.05; ^Δ^There was a significant statistical difference, compared to the NCI group, P < 0.05.

PVWMH, periventricular white matter hyperintensities; DWMH, deep white matter hyperintensities; S-CC WMH, the splenium of the corpus callosum WMH; ES_Chisquaretest_ = V = $\sqrt{\frac{\chi 2}{N\times \min\left(r-1,c-1\right)}}$ (N: total sample size), min(r – 1, c – 1): the smaller value between number of rows (r) minus 1 and number of columns (c) minus 1; ES_ANOVAtest_ = η^2^ = $\frac{SS_{between}}{SS_{total}}$ (SS_between_: between-Groups Sum of Squares, SS_total_, total Sum of Squares); ES _Htest_= adjusted ε^2^ = $\frac{H-\left(k-1\right)}{N-1}$ (k: number of groups, N: total sample size).

### 3.2 Hemodynamics parameters

**Figure 4:**
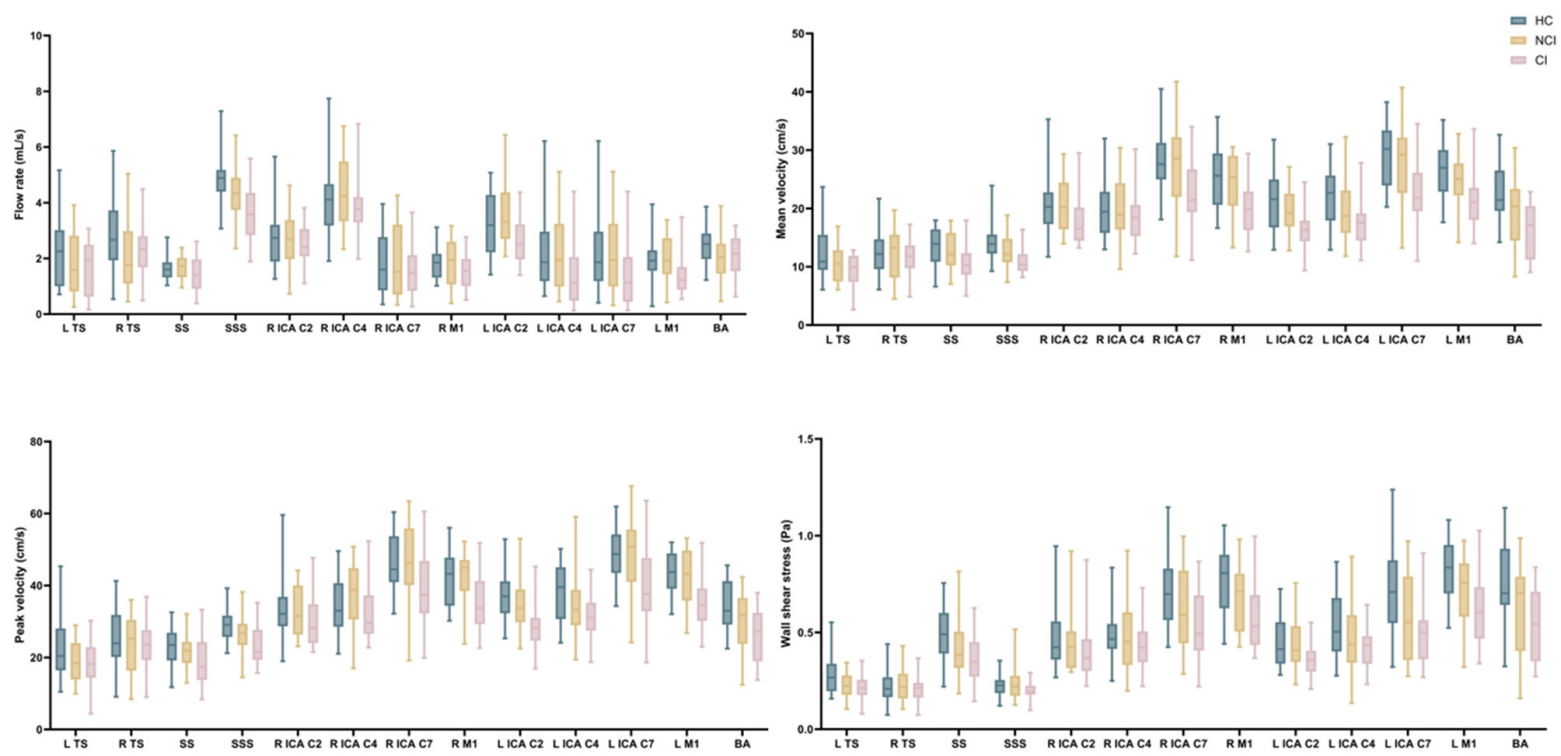
Comparison of hemodynamic parameters of arterial segments and venous sinuses between CSVD patients and HCs. TS, transverse sinus; SS, straight sinus; SSS, superior sagittal sinus; ICA, internal carotid artery; M1, middle cerebral artery M1 segments; BA, basilar artery.

**Figure 5:**
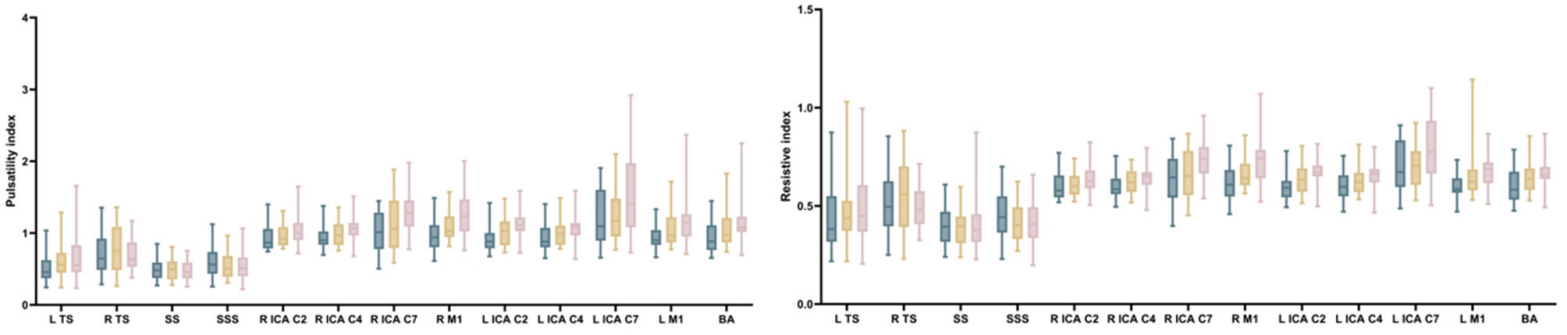
Comparison of hemodynamic parameters of arterial segments and venous sinuses between CSVD patients and HCs. TS, transverse sinus; SS, straight sinus; SSS, superior sagittal sinus; ICA, internal carotid artery; M1, middle cerebral artery M1 segments; BA, basilar artery.

Results for the hemodynamic analysis are summarized for all vessel segments in Figures 4, 5 and Supplemental file.

**NCI group:** Compared with the HC group, the NCI group exhibited statistically significant decreased flow rate and WSS (flow rate: right TS and BA segments; WSS: left TS, left ICA C7 and BA segments); No significant differences were found for the cross-sectional area, mean velocity, peak velocity, PI, and RI in all vessel segments of interest.

**CI group:** Compared with the HC group, the cross-sectional area of the BA, the PI and RI of the ICA, MCA (PI: left: C2, C4, M1 segments; right: C4, C7, M1 segments, RI: left: C2, C4, C7, M1 segments; right: C4, C7, M1 segments) and BA was statistically increased in the CI group; The flow rates of the SSS, left ICA and MCA (C2, C4, C7, M1 segments) and BA segments, the mean and peak velocity of SS, SSS, BA, ICA and MCA (left: C2, C4, C7, M1 segments; right: C7, M1 segments), the WSS of left TS, SS, BA, ICA and MCA (left: C2, C4, C7, M1 segments; right: C7, M1 segments) exhibited a statistically significant decrease in the CI population. No significant difference was found for the PI and RI in all venous sinuses, and the PI and RI of all left arterial segments, right arterial segments except the ICA C2, and BA showed a statistically significant increase in the CI group when compared with the HC group.

No significant difference was found in cross-sectional area across all vessel segments of interest, as well as in WSS and PI in all venous sinuses between the CI and NCI groups. Compared with the NCI group, the flow rate in the left arterial segments, except the ICA C7, showed a statistically significant decrease in the CI group. For mean velocity and peak velocity, the SS, ICA and MCA (left: C2, C7, M1 segments; right: C7, M1 segments) had a statistically significant decrease in the CI group. Regarding WSS, the left ICA C2 and bilateral M1 segments had a statistically significant decrease in the CI group, with the RI of the left ICA C7 in CI group higher than that in the NCI group.

### 3.3 CSF fluid dynamics

Results for the CSF dynamics analysis are presented in Table 2.

**Table 2:** Comparison of CSF fluid dynamics at midbrain aqueduct.

**CSF fluid dynamics parameters**	**HC (*N =* 25)**	**NCI (*N =* 23)**	**CI (*N =* 30)**	***P*_HC − NCI_**	***P*_HC − CI_**	***P*_NCI − CI_**	**ES**	***P***
Area	0.03 (0.03,0.04)	0.03 (0.03,0.04)	0.04 (0.03,0.05)	1.000	0.056	0.096	0.065	–
Flux-FH	0.07 (0.05,0.10)	0.10 (0.06,0.14)	0.10 (0.08,0.13)	0.037^*^	0.001^*^	1.000	0.149	–
Flux-HF	−0.11 (−0.13, −0.07)	−0.12 (−0.19, −0.09)	−0.13 (−0.17, −0.11)	0.077	0.022^*^	1.000	0.081	–
Mean Velocity-FH	1.91 (1.44, 2.71)	2.72 (1.79, 3.36)	2.61 (2.13, 3.04)	0.044^*^	0.049^*^	1.000	0.075	–
Mean velocity-HF	−2.66 (−3.37, −2.05)	−3.48 (−4.16, −2.95)	−2.78 (−4.35, −2.46)	0.025^*^	0.430	0.577	0.065	–
Peak velocity	4.06 (3.35,4.99)	5.22 (3.66,5.92)	4.62 (3.96,6.02)	–	–	–	0.049	0.056

^*^There was a significant statistical different, P < 0.05.

FH, foot to head side; HF, head to foot side; ES _Htest_ = adjusted ε^2^ = $\frac{H-\left(k-1\right)}{N-1}$ (k: number of groups, N: total sample size).

**NCI group:** The NCI group exhibited a statistically increase of the flux-FH, mean velocity in both direction when compared with the HC group, while no significant difference was found for the area and peak velocity of the midbrain aqueduct.

**CI group:** The CI group exhibited a statistically increase of the flux in both direction, and mean velocity-FH when compared with the HC group, while no significant difference was found for the area and peak velocity of the midbrain aqueduct.

### 3.4 Spearman correlation analysis: hemodynamics and cognitive impairment

**Figure 6:**
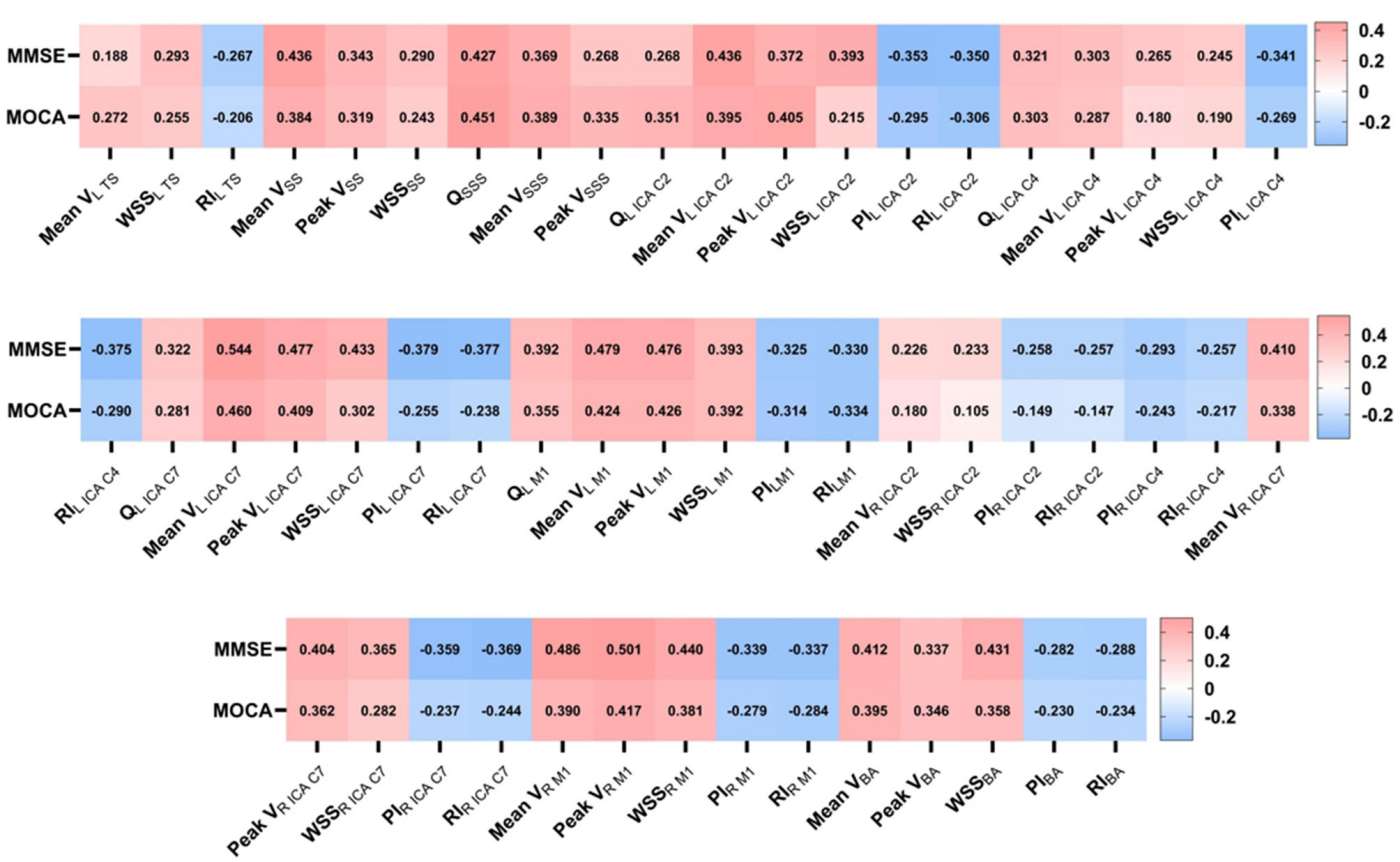
The heat map of correlation analysis of hemodynamic parameters with cognitive assessment (MMSE, MoCA). The number in the box represented the statistically significant correlation coefficient (*r* value). Mean V, mean velocity; Peak V, peak velocity; TS, transverse sinus; SS, straight sinus; SSS, superior sagittal sinus; ICA, internal carotid artery; M1, middle cerebral artery M1 segments; BA, basilar artery.

As show in Figure 6, there was a moderately positive correlation between MMSE, MoCA score, and flow rate, mean velocity, peak velocity and WSS (*r* = 0.105–0.544, all *P* < 0.05), and a moderately negative correlation between MMSE, MoCA score and PI, RI (*r* = −0.206 to −0.379, all *P* < 0.05) in some specific arterial segments and venous sinuses of interest included in this study. For the flow rate, the correlation coefficient between MoCA score and flow rate in the SSS was the highest (*r* = 0.451, *P* < 0.001), while the correlation between MMSE score and flow rate in the left ICA C2 segment was the weakest (*r* = 0.268, *P* = 0.020). For blood-flow velocity, the correlation coefficient between MMSE score and mean velocity in the left ICA C7 segment was the highest (*r* = 0.544, *P* < 0.001), correlation between MMSE score and mean velocity in the right ICA C2 segment was the weakest (*r* = 0.226, *P* = 0.049). For the WSS, the correlation coefficient between MMSE score and WSS in the left ICA C7 segment was the highest (*r* = 0.433, *P* < 0.001), while the correlation between MMSE score and WSS in the right ICA C2 segment was the weakest (*r* = 0.233, *P* = 0.043). For the PI, the correlation coefficient between MMSE score and PI in the right ICA C7 segment was the highest (*r* = −0.406, *P* < 0.001), whereas the correlation between MoCA score and PI in the BA segment was the weakest (*r* = −0.230, *P* = 0.047). For the RI, the correlation coefficient between MMSE score and RI in the left ICA C7 segment was the highest (*r* = −0.377, *P* = 0.001), while the correlation between MoCA score and RI in the BA segment was the weakest (*r* = −0.234, *P* = 0.043).

### 3.5 Multiple linear regression: hemodynamics and WMH

Multiple linear regression was conducted to investigate whether the hemodynamic parameters of each vascular segment were possible factors independently associated with the degree and distribution of WMH (Table 3). Fazekas grade, PVWMH, DWMH, and S-CC WMH were treated as separate dependent variables in their corresponding models. The flow rate, mean velocity, WSS, PI of each vascular segment were separately included as independent variables in every model. The covariates of all models included age, sex, BMI, education years, vascular risk factors, and unhealthy habits, to exclude the interference of these confounding factors. The results indicated that, after adjustment for covariates, the flow rate and mean velocity in venous sinuses (β = −0.472 to −0.381, all *P* < 0.05) and the WSS in arterial segments (β = −0.771 to −0.373, all *P* < 0.05) had significantly and independently negative associations with Fazekas grade, PVWMH, DWMH and S-CC WMH; Meanwhile, a significant positive relationship between PI in arterial segments and specific-distributed WMH (PVWMH and S-CC WMH) was found (β = 0.239 to 0.356, all *P* < 0.05), indicating that individuals with higher PI have higher degree of WMH at some specific locations.

**Table 3:** Multiple linear regression of hemodynamics and severity of WMH.

**Hemodynamics parameters**	**Vascular segments**	**WMH**	**β**	**95% CI**	***t*-value**	**Adjusted *R*^2^**	***P***
Mean velocity	SS	Fazekas grade	−0.468	(−0.488, −0.067)	−2.637	0.267	0.011
		PVWMH	−0.460	(−0.245, −0.034)	−2.635	0.288	0.011
		DWMH	−0.430	(−0.257, −0.019)	−2.314	0.194	0.024
Flow rate	SSS	DWMH	−0.381	(−0.667, −0.036)	−2.227	0.252	0.030
		S-CC WMH	−0.472	(−0.884, −0.115)	−2.596	0.152	0.012
WSS	R ICA C7	Fazekas grade	−0.633	(−9.346, −2.328)	−3.331	0.311	0.002
		PVWMH	−0.509	(−4.200, −0.589)	−2.656	0.301	0.010
		DWMH	−0.703	(−5.330, −1.556)	−3.654	0.294	0.001
	R M1	Fazekas grade	−0.699	(−10.62, −3.503)	−3.979	0.398	< 0.001
		PVWMH	−0.563	(−4.890, −1.017)	−3.059	0.339	0.003
		DWMH	−0.771	(−5.991, −2.225)	−4.374	0.395	< 0.001
		S-CC WMH	−0.555	(−6.106, −1.207)	−2.816	0.244	0.007
	L ICA C7	Fazekas grade	−0.554	(−8.158, −1.585)	−2.967	0.343	0.004
		PVWMH	−0.435	(−3.717, −0.242)	−2.280	0.313	0.026
		DWMH	−0.625	(−4.640, −1.145)	−3.313	0.328	0.002
		S-CC WMH	−0.373	(−4.116, −0.013)	−2.014	0.353	0.049
PI	R ICA C4	PVWMH	0.261	(0.043, 2.742)	2.063	0.221	0.043
	L ICA C2	PVWMH	0.259	(0.008, 2.521)	2.013	0.342	0.049
		S-CC WMH	0.356	(0.570, 3.641)	2.742	0.329	0.008
	L ICA C4	PVWMH	0.243	(0.007, 2.508)	2.009	0.328	0.049
		S-CC WMH	0.304	(0.399, 3.397)	2.531	0.284	0.014
	L ICA C7	S-CC WMH	0.304	(0.107, 0.987)	2.487	0.353	0.016
	L M1	PVWMH	0.239	(0.044, 1.197)	2.157	0.384	0.035
		S-CC WMH	0.247	(0.077, 1.493)	2.221	0.384	0.030

SS, straight sinus; SSS, superior sagittal sinus; ICA, internal carotid artery; M1, middle cerebral artery M1 segments; WSS, wall shear stress; PI, pulsatility index; β, standardized coefficient; 95% CI, 95% confidence interval.

## 4 Discussion

Cerebral small vessel disease is a progressive microvascular disorder that significantly impacts brain health in aging populations (Cannistraro et al., 2019; Huang et al., 2022). While its clinical manifestations are heterogeneous, emerging evidence highlights the critical role of hemodynamic dysfunction in disease progression (Berman et al., 2017; van Dinther et al., 2023). In this study, we employed advanced 4D flow MRI and 2D PC MRI techniques to characterize alterations in cerebral arterial/venous hemodynamics and CSF dynamics in CSVD patients with CI, aiming to elucidate their pathophysiological contributions beyond conventional structural imaging findings.

Previous studies have suggested that increased arterial stiffness may be related to an increase in arterial lumen diameter (Watanabe et al., 1996), however, this has been disputed by others. In our current study, a statistically significant increase in the vascular cross-sectional area was observed only in the basilar artery when comparing the CI and HC groups. The results of vessel area analysis in this study should be interpreted with caution, because the accuracy of vascular cross-sectional area calculation is limited by the spatial resolution of the MRI sequence. CSVD is a dynamic whole-brain disease (Wardlaw et al., 2019). As the disease progresses, cerebral blood flow diminishes throughout the entire brain and in most specific regions, resulting in increasingly severe damage, irrespective of the lesion's location. Vascular endothelial dysfunction and impaired self-regulation are the main pathological mechanisms of CSVD, which can result in microcirculation disorders, particularly in capillary flow, and damage to the permeability of the blood-brain barrier (BBB) (Wardlaw et al., 2013a; Erdener and Dalkara, 2019; Caplan, 2015). However, transport through the BBB and perivascular drainage are the primary pathways for waste metabolism in the brain. Our results demonstrated that the flow rate and velocity (including mean velocity and peak velocity) had a moderately positive correlation with MMSE and MoCA score, and compared with the HC group, exhibited a statistically significant decrease in the CSVD patients with CI. Low hemodynamics may cause the cerebral hypoperfusion and subsequent intermittent hypoxia provoke oxidative stress, mitochondrial dysfunction, inflammation, and proteinopathy, leading to neurodegeneration (Burtscher et al., 2021). Zhang et al. (2021) reported that decreased cerebral blood flow was closely related to more severe cognitive impairment or faster cognitive decline rate. Also, the hypoperfusion reduces capillary pericyte coverage and disrupts BBB integrity, which will aggravate plasma protein leakage and leukocyte infiltration into the brain parenchyma, leading to glial cell activation, demyelination and neurodegeneration (Inoue et al., 2023). Rivera-Rivera et al. found that the average blood flow in all arterial segments of patients with mild cognitive impairment (MCI) were decreased (Rivera-Rivera et al., 2015), which is consistent with our results. The decrease in vascular drainage indicates a weakening of the vascular “pumping” mechanism, which is the main driving force for metabolic waste clearance (Arbel-Ornath et al., 2013). The failure to process soluble metabolites, such as cerebral amyloid (CA), in the brain is significant for the pathogenesis of CI (Saito et al., 2015). CA deposition has been associated with loss of smooth muscle cells, thickening of the basement membrane, narrowing of the vascular lumen, loss of cerebrovascular autoregulation, decreased vascular reactivity, and increased incidence of microscopic infarcts. These pathological changes delay the drainage of interstitial fluid, which in turn promotes CA aggregation and cognitive decline (Arbel-Ornath et al., 2013). Venous collagenosis is another important feature of CSVD, which can lead to intramural thickening, stenosis, and ultimately luminal occlusion, resulting in elevated venous pressure and a reduction in blood flow velocity in the venous system (Zhang et al., 2022). We found that mean and peak velocity of venous sinuses (SS and SSS) were lower in CSVD patients.

WSS is the friction force generated by the flow of viscous blood parallel to the vessel wall. Reduced or oscillating WSS is associated with local endothelial dysfunction and atherosclerosis (Zhou et al., 2014; Kwak et al., 2014). Liu et al. (2016) found that the mean and peak carotid WSS were independently associated with WMH and MMSE score after adjusting for confounding factors, which indicated that changes in local rheologic forces may contribute to the important effects on white matter lesions and cognitive impairment in older patients. Our study showed that the CSVD with CI population had lower WSS in cerebral arteries and venous sinuses and was associated with the poor cognitive status. Low ICA and BA WSS have been proved to be related with the CI and Alzheimer's disease (AD) (van Es et al., 2009). Previous study found that patients with AD and MCI had significantly decreased carotid systolic, diastolic and mean WSS than cognitively healthy participants (van Es et al., 2009). Intracranial PI is an indicator of increased distal resistance of blood flow in microvessels, which is usually associated with CSVD (Rodríguez et al., 2010). The resistive index (RI), although mathematically similar to PI, provides information regarding the microvascular bed distal to the site of measurement and offers another parameter to characterize cerebrovascular flow features (Rivera-Rivera et al., 2015). Pahlavian et al. (2021) found that elevated RI was significantly associated with reduced cognitive performance, additionally, PI and RI were both significantly associated with relative WMH volume. Increased pulsatility of cerebral arteries has been reported in CSVD patients (Shi et al., 2018b) and to be associated with cognitive decline, which supported for our findings. Also, the increased cerebroarterial pulsation has been suggested to cause perivascular shear stress and damage to oligodendrocytes causing dysfunction of perivascular glymphatic system (Pahlavian et al., 2021). The clinical manifestation of these structural damages has been demonstrated in reports that associated WMH with CI (Murray et al., 2010). Additionally, the increased flux and velocity of CSF in the midbrain adequate were observed in the CSVD with CI patients in this study. The arterial pulse wave drives CSF and venous blood to flow out of the cranial cavity. Therefore, CSF in and out of the cranial cavity helps to reduce net brain volume change. Increased arterial pulsation can be compensated to a certain extent by the increase in CSF pulsation (Stoquart-ElSankari et al., 2007). There is interdependence between changes in blood flow and CSF flow. Under conditions of arteriovenous drainage disorder, the movement of cerebrospinal fluid is impaired, leading to an increase in CSF pressure relative to the peak value of arterial load at certain stages of the cardiac cycle. This is reflected in changes in flow and velocity, which may affect the formation of atrophy and WMH (Dobrynina et al., 2020).

Recently, a study found that WMH in the splenium of the corpus callosum (S-CC WMH) is closely related to cognitive decline, suggesting that WMH in specific local brain regions is more correlated with cognitive function and may be used as a potential imaging marker for CI (Garnier-Crussard et al., 2021). Therefore, based on previous findings, and after excluding confounding factors such as age, gender, and vascular risk factors, the current study established multiple linear regression models to further explore the relationship between hemodynamics and the severity and distribution of WMH. For patients with CSVD, the deposition of abnormal substances in small arteries can lead to stiffness and stenosis of the lumen, thereby increasing vascular resistance and reducing blood perfusion in white matter, resulting in the development of WMH (Gouw et al., 2010; Diamond, 2016; Jensen et al., 2017). A recent study that combined brain histopathological with brain MRI demonstrated that collagen deposition in veins is positively correlated with WMH volume (Lahna et al., 2022), which may also damage the hemodynamics of the intracranial venous sinus, resulting in decreased flux and blood flow velocity. We found that the blood flow rate and velocity in venous sinuses had significantly and independently negative associations with WMH. In addition to systemic risk factors such as age and genetic variation, vascular pathophysiological factors such as arterial WSS are increasingly thought to be important contributors to chronic cerebrovascular disease (Nixon et al., 2010; Chen et al., 2019). Low WSS is closely associated with endothelial failure (Papafaklis et al., 2015; Kwak et al., 2014)and has an independently association with WMH volume and fraction, as well as the progression of lacuna and microbleeds in the elderly. A previous study indicated that the risk of new incidents of Fazekas scale ≥2 lesions was significantly higher in the lowest WSS quartile group compared to those in the higher WSS quartile group (Aribisala et al., 2014). Our study found that arterial WSS had a significant and independent negative correlation with WMH and was not related to its distribution. In other words, whether in the whole brain or in specific brain regions, individuals with lower arterial WSS may exhibit more severe WMH. Increasing stiffness of the large central arteries is associated with WMH. One explanation for the association between arterial stiffness and WMH is that arterial stiffening exposes small vessels in the brain to high pulsatility, damaging the small vessel walls (Aribisala et al., 2014). As mentioned above, PI can quantify vascular pulsation and distal blood flow resistance. Our results indicated that arterial PI was significantly positively correlated with the degree of PVWMH and S-CC WMH. Traub et al. (2023) suggested that the proximal cerebral artery constricts in chronic heart failure, which alters vascular PI and may promote the development of WMH by reducing blood flow to arteriosclerotically damaged arterioles and dysfunctional capillary endothelium. A study involving 694 community residents indicated that WMH was associated with a higher internal carotid artery PI after adjusting for blood pressure and correcting for multiple tests, as our results showed (Traub et al., 2023).

As a prospective study, our research has several limitations. Firstly, it involves a small sample size and conducted at a single center. Therefore, selection bias may have influenced the results, ideally, the distribution of participants among the three groups should be more uniform, and due to the limited sample size, we did not conduct a more detailed stratification of the CSVD subtype. Further research will be carried out to potentially addressing the inherent heterogeneity of CSVD, which may provide more nuanced insights. Secondly, the collection of vascular risk factors and unhealthy habits of the participants is not comprehensive. For example, we only recorded whether the participants had hyperlipidemia, but did not record the specific indicators such as total cholesterol and low-density lipoprotein in detail. Additionally, only dementia screening measures were used for cognition assessment, there is no insight into the domains, extent, or true magnitude of impairment. Thirdly, for the evaluation of WMH, we used traditional visual scoring methods, specifically the Fazekas classification, instead of quantitative measurement techniques such as volume segmentation of 3D sequences. The subjectivity of the observer may affect our results. Fourthly, due to limitations in time and spatial resolution, small vessels with slow blood flow may experience signal loss on 4D flow MRI images, which has a negative impact on hemodynamic analysis. Finally, the cross-sectional study design limits the ability to infer causality. Future longitudinal studies should be conducted to validate these findings and explore temporal relationships.

In conclusion, this study demonstrates the feasibility of using 4D flow MRI to analyze the hemodynamics of arterial and venous regions with larger lumens in the context of CSVD. It establishes the relationship between hemodynamics and cognitive function and white matter high signal. This research helps identify patients who may benefit from interventions to improve cerebral circulatory system functions and provides a potential new avenue for clinical treatment.

## Data Availability

The raw data supporting the conclusions of this article will be made available by the authors, without undue reservation.
