# Supplementary material for: Hemodynamic changes and their relationship with white matter hyperintensities in CSVD patients with cognitive impairment: a 4D flow study
Source: Front Aging Neurosci. 2025 Jun 18;17:1578288. doi: 10.3389/fnagi.2025.1578288 (PMC12213705; doi:10.3389/fnagi.2025.1578288)
Supplement: Supplementary file 1 [file Data_Sheet_1.pdf]

		HC (n=25)	NCI (n=23)	CI (n=30)	P_{HC-NCI}	P_{HC-CI}	P_{NCI-CI}	P
Area(mm^2)	L TS	30.71±15.00	30.51±13.44	27.07±10.69	-	-	-	0.636
	R TS	39.66±13.81	33.91±11.64	39.77±10.18	-	-	-	0.160
	SS	13.15±2.67	14.59±4.10	14.64±4.10	-	-	-	0.278
	SSS	38.40±5.25	39.48±8.98	38.68±5.62	-	-	-	0.848
	L ICA C2	28.20±6.31	30.16±8.26	28.45±5.36	-	-	-	0.559
	L ICA C4	26.46±5.70	27.77±7.01	26.01±5.36	-	-	-	0.571
	L ICA C7	19.99±3.88	20.34±5.02	19.77±4.99	-	-	-	0.912
	L M1	12.13±1.99	13.48±2.19	12.22±2.83	-	-	-	0.127
	R ICA C2	24.22±6.09	26.20±6.42	26.11±5.70	-	-	-	0.429
	R ICA C4	24.16(20.00,26.73)	26.92(21.14,31.27)	24.15(21.22,28.35)	-	-	-	0.306
	R ICA C7	16.67±3.87	18.41±5.54	19.31±5.24	-	-	-	0.179
	R M1	12.21(10.31,13.83)	14.13(12.12,16.33)	12.80(11.57,15.71)	-	-	-	0.082
	BA	14.49(11.84,15.30)	13.93(11.76,17.49)	15.85(14.35,18.98)	1.000	0.036*	0.141	-
Flow rate (mL/s)	L TS	2.38(1.00,3.19)	1.59(0.82,2.81)	1.97(0.63,2.49)	-	-	-	0.173
	R TS	2.84±1.29	2.07±1.27	2.32±0.94	0.028*	0.109	0.439	-
	SS	1.62±0.40	1.69±0.39	1.45±0.62	-	-	-	0.176
	SSS	4.89(4.40,5.17)	4.34(3.75,4.90)	3.58(2.84,4.35)	0.158	<0.001*	0.105	-
	L ICA C2	3.26±1.10	3.58±1.06	2.61±0.84	0.284	0.019*	0.001*	-
	L ICA C4	4.81±1.19	4.60±1.19	3.78±1.18	0.535	0.002*	0.017*	-
	L ICA C7	1.87(1.18,2.96)	1.95(0.99,3.24)	1.13(0.48,2.05)	1.000	0.049*	0.082	-
	L M1	1.88(1.45,2.29)	1.91(1.43,2.72)	1.23(0.88,1.69)	1.000	0.030*	0.013*	-
	R ICA C2	2.69±0.97	2.72±0.97	2.48±0.75	-	-	-	0.567
	R ICA C4	4.11(3.18,4.67)	4.23(3.33,5.48)	3.77(3.30,4.20)	-	-	-	0.305
	R ICA C7	1.60(0.86,2.77)	1.52(0.70,3.21)	1.48(0.84,2.11)	-	-	-	0.800
	R M1	1.85±0.58	1.90±0.84	1.56±0.60	-	-	-	0.179
	BA	2.50±0.68	2.04±0.85	2.07±0.73	0.040*	0.041*	0.893	-
Mean velocity (cm/s)	L TS	10.89(9.45,15.46)	10.57(7.50,12.85)	9.96(7.42,11.86)	-	-	-	0.248
	R TS	12.94±4.63	12.20±4.20	11.99±2.91	-	-	-	0.662
	SS	13.48±3.10	12.73±2.96	10.62±2.88	0.392	0.001*	0.012*	-

	SSS	13.95(12.24,15.53)	12.16(10.76,14.78)	10.36(9.28,12.17)	0.201	<0.001*	0.083	-
	L ICA C2	20.99±4.84	19.62±3.63	16.14±3.14	0.239	<0.001*	0.003*	-
	L ICA C4	21.62±5.07	19.67±5.03	17.20±3.58	0.147	0.001*	0.058	-
	L ICA C7	29.16±5.21	27.77±6.76	22.36±5.42	0.429	<0.001*	0.002*	-
	L M1	26.50±4.37	24.54±5.04	21.51±4.48	0.173	<0.001*	0.027*	-
	R ICA C2	20.26(17.33,22.76)	20.21(16.43,24.42)	16.52(14.46,20.15)	1.000	0.075	0.171	-
	R ICA C4	19.84±4.92	20.14±5.50	18.51±4.39	-	-	-	0.444
	R ICA C7	27.85±5.65	27.45±7.61	22.65±5.50	0.837	0.005*	0.010*	-
	R M1	25.62(20.61,29.43)	25.39(20.46,29.03)	19.86(16.29,22.89)	1.000	0.006*	0.032*	-
	BA	21.43(19.56,26.50)	20.36(14.49,23.34)	17.11(11.23,20.37)	0.272	<0.001*	0.124	-
Peak velocity (cm/s)	L TS	22.72±9.06	18.90±5.87	17.85±6.43	-	-	-	0.103
	R TS	24.68±8.73	23.36±8.26	23.39±6.38	-	-	-	0.879
	SS	23.54±5.37	21.94±4.54	18.79±6.05	0.315	0.002*	0.040*	-
	SSS	29.08(25.71,31.60)	26.83(23.47,29.36)	21.55(19.35,27.68)	0.375	0.001*	0.083	-
	L ICA C2	36.82±7.02	34.79±7.02	28.41±5.66	0.297	<0.001*	0.001*	-
	L ICA C4	38.05±8.43	34.44±8.66	31.40±6.08	0.113	0.002*	0.165	-
	L ICA C7	49.13±7.10	48.18±11.29	39.18±10.45	0.750	0.001*	0.002*	-
	L M1	43.33±6.00	41.76±8.55	35.54±7.21	0.489	<0.001*	0.004*	-
	R ICA C2	32.17(28.68,36.84)	31.53(26.36,40.02)	28.25(24.05,34.75)	-	-	-	0.132
	R ICA C4	33.01(28.53,40.65)	38.84(30.56,44.80)	29.66(26.58,37.27)	-	-	-	0.104
	R ICA C7	46.05±7.92	45.70±11.24	39.16±9.56	0.906	0.015*	0.022*	-
	R M1	43.24(34.35,47.75)	45.04(38.45,47.09)	33.70(29.28,41.24)	1.000	0.016*	0.009*	-
	BA	34.66±6.52	30.43±8.40	26.41±7.53	0.057	<0.001*	0.064	-
WSS(Pa)	L TS	0.28±0.11	0.22±0.07	0.22±0.07	0.040*	0.025*	0.836	-
	R TS	0.22±0.09	0.23±0.09	0.21±0.06	-	-	-	0.672
	SS	0.49±0.14	0.42±0.17	0.37±0.12	0.106	0.003*	0.178	-
	SSS	0.23(0.19,0.25)	0.22(0.17,0.28)	0.20(0.18,0.22)	-	-	-	0.162
	L ICA C2	0.46±0.14	0.43±0.14	0.35±0.08	0.450	0.002*	0.022*	-
	L ICA C4	0.54±0.18	0.47±0.19	0.42±0.10	0.165	0.006*	0.213	-

	L ICA C7	0.71±0.22	0.59±0.23	0.50±0.16	0.039*	<0.001*	0.120	-
	L M1	0.82±0.15	0.72±0.19	0.61±0.16	0.054	<0.001*	0.026*	-
	R ICA C2	0.42(0.36,0.56)	0.43(0.32,0.51)	0.37(0.30,0.47)	-	-	-	0.165
	R ICA C4	0.47(0.42,0.54)	0.45(0.33,0.60)	0.42(0.35,0.51)	-	-	-	0.281
	R ICA C7	0.71±0.19	0.62±0.21	0.53±0.18	0.147	0.002*	0.103	-
	R M1	0.78±0.16	0.68±0.18	0.57±0.16	0.064	<0.001*	0.028*	-
	BA	0.76±0.19	0.62±0.25	0.53±0.19	0.022*	<0.001*	0.159	-
PI	L TS	0.46(0.37,0.68)	0.57(0.46,0.75)	0.59(0.47,0.86)	-	-	-	0.314
	R TS	0.65(0.49,0.92)	0.77(0.49,1.19)	0.63(0.53,0.87)	-	-	-	0.611
	SS	0.48(0.37,0.59)	0.50(0.36,0.61)	0.46(0.37,0.61)	-	-	-	0.947
	SSS	0.61±0.25	0.55±0.19	0.55±0.21	-	-	-	0.478
	L ICA C2	0.93±0.18	1.02±0.19	1.11±0.19	0.107	0.001*	0.091	-
	L ICA C4	0.93±0.17	1.00±0.18	1.07±0.19	0.206	0.008*	0.196	-
	L ICA C7	1.09(0.90,1.61)	1.17(0.95,1.48)	1.40(1.10,1.97)	-	-	-	0.058
	L M1	0.88(0.80,1.04)	0.97(0.87,1.22)	1.15(0.95,1.26)	0.314	0.007*	0.549	-
	R ICA C2	0.87(0.79,1.06)	0.92(0.83,1.08)	1.00(0.90,1.14)	-	-	-	0.115
	R ICA C4	0.94±0.17	1.00±0.18	1.05±0.19	0.207	0.019*	0.322	-
	R ICA C7	1.01(0.78,1.29)	1.06(0.79,1.45)	1.28(1.10,1.47)	1.000	0.010*	0.140	-
	R M1	0.97(0.81,1.21)	1.04(0.95,1.26)	1.27(1.04,1.47)	0.826	0.010*	0.245	-
	BA	0.88(0.76,1.11)	0.98(0.87,1.21)	1.08(1.02,1.23)	0.233	0.024*	1.000	-
RI	L TS	0.38(0.32,0.55)	0.44(0.37,0.53)	0.45(0.37,0.61)	-	-	-	0.448
	R TS	0.51±0.16	0.55±0.18	0.49±0.10	-	-	-	0.407
	SS	0.39(0.32,0.47)	0.40(0.31,0.45)	0.38(0.32,0.46)	-	-	-	0.875
	SSS	0.46±0.14	0.42±0.11	0.42±0.11	-	-	-	0.396
	L ICA C2	0.60±0.07	0.63±0.07	0.67±0.07	0.085	<0.001*	0.079	-
	L ICA C4	0.60±0.07	0.63±0.07	0.65±0.07	0.191	0.007*	0.189	-
	L ICA C7	0.70±0.13	0.71±0.11	0.79±0.15	0.741	0.018*	0.041*	-
	L M1	0.58(0.57,0.64)	0.62(0.58,0.69)	0.69(0.62,0.72)	0.516	0.008*	0.340	-
	R ICA C2	0.58(0.54,0.65)	0.60(0.56,0.65)	0.62(0.59,0.68)	-	-	-	0.137

	R ICA C4	0.60±0.07	0.63±0.06	0.64±0.07	0.190	0.014*	0.292	-
	R ICA C7	0.65(0.54,0.74)	0.65(0.55,0.78)	0.74(0.67,0.80)	1.000	0.011*	0.127	-
	R M1	0.61(0.55,0.70)	0.64(0.61,0.72)	0.74(0.64,0.79)	0.817	0.008*	0.221	-
	BA	0.60±0.09	0.65±0.08	0.66±0.08	0.055	0.013*	0.648	-
